# Maternal fat free mass during pregnancy is associated with birth weight

**DOI:** 10.1186/s12978-017-0308-3

**Published:** 2017-03-28

**Authors:** Yanxia Wang, Jie Mao, Wenling Wang, Jie Qiou, Lan Yang, Simin Chen

**Affiliations:** 1 Institute of Maternity and Child-Care Research, Gansu Provincial Maternity and Child-care Hospital, No.143.Qilihe north Rd., Lanzhou, Gansu 730030 People’s Republic of China; 2 Department of Nutrition, Lanzhou University the Second hospital, Lanzhou, Gansu China

**Keywords:** Birth weight, Maternal body composition, Fat free mass, Fat mass

## Abstract

**Background:**

The relationship between maternal body compositions and birth weight was not definite. Fat Mass (FM) and Fat Free Mass (FFM) can accurately reflect the maternal body fat compositions and have been considered as better predictors of birth weight. Despite its potential role, no studies have been described the maternal compositions during pregnancy in East Asian women previously. We investigated the correlation between birth weight and Maternal body composition including fat mass (FM) and fat free mass (FFM). To determine whether birth weight is associated with maternal body fat FM and FFM during pregnancy and, if so, which trimester and parameter is more critical in determining birth weight.

**Methods:**

A longitudinal prospective observational study performed, 348, 481 and 321 non-diabetics Han Chinese women with a singleton live birth attending a routine visit in their first, second and third trimesters were recruited. Maternal body composition was measured using segmental multi-frequency bioelectrical impedance analysis. Data of the pre-pregnancy body mass index (BMI), maternal BMI, the gestational weight gain (GWG), and placental and birth weight were collected.

**Results:**

A significant correlation exists between maternal FFM in the process of pregnancy, placental weight, GWG at delivery, and birth weight (*P* < 0.05). On stepwise multiple linear regression analysis, material’s FFM was the most important factor associated with the birth weight. After adjustment, there was significantly associated with 2.47-fold increase in risk for birth weight more than 4 kg when FFM ≥ 40.76 kg (Upper quartile of participants). The increased maternal age became a protective factor (OR = 0.69) while the increased pre-pregnancy BMI (OR = 1.50) remained predictors to birth weight more than 4 kg.

**Conclusions:**

The change of maternal FFM during pregnancy is independently affected the birth weight.

## Plain English summary

The relationship between maternal body compositions and birth weight was not definite. Fat Mass (FM) and Fat Free Mass (FFM) can accurately reflect the maternal body fat compositions and have been considered as better predictors of birth weight. Despite its potential role, no studies have been described the maternal compositions during pregnancy in East Asian women previously. We investigated the correlation between birth weight and Maternal body composition including fat mass (FM) and fat free mass (FFM).

A longitudinal prospective observational study was performed. 348, 481 and 321 non-diabetics Han Chinese women with a singleton live birth attending a routine visit in their first, second and third trimesters were recruited respectively at the Gansu Provincial Maternity & Child Care Hospital, China. Maternal body composition (including FM and FFM) was measured using segmental multi-frequency bioelectrical impedance analysis.

The prenatal testing records of the pre-pregnancy body mass index (BMI) and maternal BMI at the tested time were collected. At delivery the gestational weight gain (GWG), placental and birth weight were weighed. Pearson correlation and multiple linear regressions were used to identify the strongest predictors of birth weight.

In conclusion, the change of maternal FFM during pregnancy is independently affected the birth weight.

## Background

There has been a growing concern in respect to birth weight because of the fetal origin hypothesis of adulthood chronic diseases [1]. Birth weight has been linked to obesity, cardiovascular diseases, diabetes, and cancer in later life [2–5]. Moreover, low birth weight is associated with fetal growth restriction or prematurity, and increases the risk of neonatal morbidity and mortality [6], while high birth weight is related to an increased risk of obstetric complications, such as dystocia, postpartum hemorrhage, and infection [7]. Maternal nutritional status plays a critical role in birth weight [8–10].

Maternal increased body mass index (BMI) and gestational weight gain (GWG) reflect pregnancy nutritional status. Excess GWG has been proved to be associated with an increasing risk of large for gestational age (LGA) [10]. Maternal weight gain include growth of body fat mass (FM), fat free mass (FFM), total body water (TBW), red blood cells mass, the fetus, placenta, amniotic fluid, and other products of conception [11]. BMI, however, is only a surrogate indicator of obesity and does not measure the distribution of fat. FM and FFM, measured by bioelectrical impedance analysis (BIA), can accurately reflect the body fat compositions and have been considered as better predictors of maternal nutritional status than BMI [11, 12].

Several epidemiologic studies have investigated the relationship between maternal body compositions and birth weight; however, results were in consistent. Some of them reported that maternal TBW associated with birth weight [13]; some found that a positive association between maternal FFM and birth weight [13–16]; some reported that maternal FM may be the major determinant of birth weight [17]. The time points of BIA measurements during pregnancy were also different in previous studies [15, 18–20]. Despite its potential role, maternal compositions during pregnancy in East Asian women have not been described previously. All these studies were only conducted in North American and European countries. In order to better understand the association between maternal body fat composition in different gestational weeks (GW) and birth weight, we conducted a study in Lanzhou, China.

## Methods

### Study design

In this longitudinal prospective observational study, the participants were randomly selected, pregnant Han Chinese women with a singleton live birth, attending a routine visit at the Center of Prenatal Care of the Gansu Provincial Maternity & Child Care Hospital (GPMCCH), the largest maternity and child care hospital in Lanzhou of China from 2012 Jun to 2014 July.

### Participants and study size

Within the 1500 participates enrolled in their search group, 1150 women who gave birth to full-term babies completed the study. 217 women were excluded because they had a multiple pregnancies (*n* = 41), a preterm birth (≤36 Weeks) (*n* = 76), GDM (*n* = 26), pregnancy-induced hypertension (*n* = 60), and other diseases which affecting maternal body weight (*n* = 19). We lost to follow-up 88 women because they changed contact information.

### Procedures

The study procedures were approved by the Human Investigation Committees at the Yale University and GPMCCH. Women who were younger than 20 years old or who were unable to provide written forms of informed consent were excluded. We also excluded women who had pre-existing diabetes mellitus, thyroid disease, pregnancy-induced hypertension diseases that affect maternal body weight later. Women with gestational diabetes mellitus (GDM) were excluded from further analysis presented for oral glucose tolerance test (OGTT) at 28^th^ gestational age. The diagnosis of GDM was made after a 2 h 75 g OGTT using the criteria outlined by the American Diabetes Association.

### Measurements

All participates had an early pregnancy ultrasound scan to confirm gestational age. At the first antenatal visit height and weight were measured digitally in a standardized way, BMI was calculated. Simultaneously, maternal body fat composition parameters were measured and recorded by foot-to-foot BIA system, which was conducted using a Body Composition Analyzer (MES-01S20, Beijing, China) with 4-point contact electrodes. The test current was 800μAand at a frequency of 50 KHz. The inter-observer coefficients for FM, FFM were 0.99 and 0.98, respectively. To ensure accurate test results, each participant was measured under the following conditions: no alcohol consumption within 24 h and no exercise or food intake during the 4 h preceding the test. All measurements were performed in the morning. Each women, wearing light clothing and without jewelry, shoes or socks, stood 1 min on the squared surface of the scale, putting the heels one per electrode and the metatarsal-phalangeal joints of each foot, one per electrode, respectively. The percentage of body fat (PBF), FM, and FFM were estimated with instrument computer software. Fat free mass index (FFMI) was analyzed by using the standard formula of FFM divided by height in meters squared.

We measured 348, 481 and 321 women in their first, second and third trimesters respectively. Based on different antenatal visit and BIA measurement time, eligible women were assigned to three study groups according to 1^st^, 2^nd^ and 3^rd^ trimesters. At delivery the GWG, placental and birth weight were weighed. The antenatal and postpartum details were obtained from the hospital’s computerized database. Maternal BMI was categorized in four groups according to the Asia-Pacific standard: the underweight group (<18.5kgm^−2^), normal weight group (18.5–22.9 kgm^−2^), overweight group (23.0–24.9 kgm^−2^) and obese group (≥25.0 kgm^−2^).

### Statistical analysis

Pearson correlations of birth weight with maternal demographic, pre-pregnancy BMI, mid-pregnancy BMI, GWG, maternal body FM and FFM, and placental weight were analyzed indifferent trimester groups. Explanatory variables identified as significant in bivariate analysis were subsequently entered into a multiple liner regression model in different trimester groups respectively, with birth weight as the dependent variable.

Multiple logistic regression analysis was then used to generate odds ratios for birth weight greater than 4 kg per BIA–measured FFM quartile, with the lowest quartile serving as the reference group. Models incorporated maternal age, gestational age, BMI, along with BIA–measured FM. *P* < 0.05 was considered for entry and removal of variables into the model.

A nominal *P* < 0.05 was considered to indicate statistical significance. Analyses were performed using statistical software package SPSS 20.0 (SPSS Inc., version 20.0, Chicago, USA).

## Results

A total of 1150 women were enrolled into the study. Among them, 348 had measured body fat composition during the first trimester, 481 during the second trimester, and 321 during the third trimester. The characteristics of the study population were presented in Table 1. The mean age was 28.0 ± 5.4 years and the mean pre-pregnancy BMI was 21.0 ± 2.8 kgm^−2^. Most of them were urban residents (92.1%) and primiparous (74.2%). Amongst the Han Chinese women studied, 13.5% were underweight, 64.6% were normal weight, 12.3% were overweight and 9.6% was obese according to the Asia-Pacific classification. The maternal BMI, FM and FFM all increased with the pregnancy process development. Among the first, second and third groups, there were no significant differences in maternal age, pre-pregnancy BMI, gestational age at delivery, and weight gain during pregnancy (*P* > 0.05).

**Table 1:** Characteristics of study popluation

N	1150
Maternal age (year)	28.0 (20.0–44.0)
Primiparous (%)	74.2
urban residents (%)	92.1
Pre-pregnancy weight (kg)	53.8 (37.1.–91.0)
Height (cm)	160.3 (143.0–178.9)
Pre-pregnancy BMI (kg m^-2^)	21.0 (15.4–32.8)
Underweight (less than 18.5, %)	13.5
Normal (18.5–22.9, %)	64.6
Overweight (23.0–24.9, %)	12.3
Obese (25.0 or higher, %)	9.6
During pregnancy	
Maternal weight gain (kg)	17.3 (9.1–38.8)
Placental weight (g)	625.1 (50.0–1000.0)
Fat mass (kg)	18.5 (8.7–54.4)
Fat-free mass (kg)	38.6 (28.9–51.2)
Fat-free mass index	15.2 (12.1–33.0)
Gestational age at delivery (weeks)	39.1 (36.5–42.0)
Mode of delivery	
Spontaneous vaginal delivery (%)	53.4
Instrumental (%)	9.2
Cesarean delivery (%)	37.4
Birth weight (g)	3370.0 (2500.0–4600.0)
Birth length (cm)	51.2 (47.0–53.9)
Neonatal male sex (%)	49.8

The Pearson correlation between birth weight and placental weight, maternal FM, FFM, pre-pregnancy BMI, mid-pregnancy BMI, and GWG was evaluated at different trimester respectively (Table 2). The significance correlations were found between birth weight and placental weight (*r* = 0.52), GWG (*r* = 0.15), maternal FM (*r* = 0.27), FFM (*r* = 0.35), pre-pregnancy BMI (*r* = 0.23), mid-pregnancy BMI (*r* = 0.23) in 1^st^ trimesters. The significance correlations were found between birth weight and placental weight (*r* = 0.55), GWG (*r* = 0.18), maternal FM (*r* = 0.28), FFM (*r* = 0.28), pre-pregnancy BMI (*r* = 0.24), mid-pregnancy BMI (*r* = 0.25) in 2^nd^ trimesters. There were significance correlations between birth weight and placental weight (*r* = 0.61), GWG (*r* = 0.15), FFM (*r* = 0.38), pre-pregnancy BMI (*r* = 0.09) in 3^rd^ trimesters.

**Table 2:** Bivariate correlations among birth weight and maternal body compositions and other related covariates in three trimesters

Variable	1	2	3	4	5	6	7	8
First trimester group (*n* = 348)								
1 Birth weight	—	0.52**	0.15*	0.23**	0.23**	0.35**	0.23**	0.27**
2 Placental weight		—	0.10	0.13	0.16*	0.27**	0.18*	0.21**
3 GWG at delivery			—	−0.29**	−0.15*	0.03**	−0.15*	−0.13
4 Pre-pregnancy BMI				—	0.92**	0.54**	0.88**	0.85**
5 Pregnancy BMI					—	0.62**	0.88**	0.85**
6 FFM						—	0.98**	0.95**
7 PBF							—	0.98**
8 FM								—
Second trimester group (*n* = 481)								
1 Birth weight	—	0.55**	0.18**	0.24**	0.25**	0.28**	0.26**	0.28**
2 Placental weight		—	0.13**	0.20**	0.21**	0.23**	0.22**	0.24**
3 GWG at delivery			—	0.12**	0.05	0.11**	0.06	0.06
4 Pre-pregnancy BMI				—	0.90**	0.54**	0.88**	0.85**
5 Pregnancy BMI					—	0.62**	0.88**	0.84**
6 FFM						—	0.68**	0.76**
7 PBF							—	0.98**
8 FM								—
Third trimester group (*n* = 321)								
1 Birth weight	—	0.61**	0.15**	0.09*	0.12	0.38**	0.15	0.20
2 Placental weight		—	0.03	0.12	0.11	0.15	0.11	0.10
3 GWG at delivery			—	0.05	0.36**	0.33**	0.43**	0.48**
4 Pre-pregnancy BMI				—	0.81**	0.34**	0.81**	0.78**
5 Pregnancy BMI					—	0.45**	0.87**	0.81**
6 FFM						—	0.56**	0.66**
7 PBF							—	0.98**
8 FM								—

Note: Correlations marked with an asterisk (*) were significant at *P* < 0.05

Correlations marked with two asterisks (**) were significant at *P* < 0.01

In multiple liner regression analysis, it was found that approximately 24% of the variation in birth weight was explained by FFM in the 1^st^ trimester, 19% was by FFM, pre-pregnancy BMI and gestational age in the 2^nd^ trimester. The associations for birth weight at the 3^rd^ trimester were weaker, with only 12% of the variation explained by FFM (Table 3). FFM were strong while FM did not contribute to the explanatory power in any significant way (*P* > 0.05).

**Table 3:** Multivariate models explaining birth weight at 1^st^, 2^nd^ and 3^rd^ trimester by use of various explanatory variables

Included variables in model	*R* ^2^	β	Standardized β	t	*P*-value	*sr*
1^st^ trimester^a^						
FFM	0.24	44.47	0.35	5.16	<0.001	0.35
2^nd^ trimester^b^						
FFM	0.19	30.72	0.22	5.83	<0.001	0.19
Pre-pregnancy BMI		16.16	0.11	2.91	0.004	0.09
Gestational age		11.35	0.07	2.24	0.025	0.07
3^rd^ trimester^c^						
FFM	0.12	68.3	0.38	3.31	0.002	0.38

Abbreviations: *R* ^2^, standardised coefficients of determination; β, unstandardised multiple regression coefficients; sr, semipartial correlations

^a^No effect of maternal age, maternal pre-pregnancy BMI, BMI at the tested time, gestational age and FM

^b^No effect of maternal age, BMI at the tested time and FM

^c^No effect of maternal age, maternal pre-pregnancy BMI, BMI at the tested time, gestational age and FM

We divided maternal FFM of participants into four quartiles: the lower quartile was 36.68 kg; the upper quartile was 40.76 kg. Maternal FM was also divided into four quartiles: the lower quartile was 14.88 kg; the upper quartile was 21.65 kg. After adjustment for age and gestational age, pre-pregnancy and mid-pregnancy BMI and FM, the maternal age (OR = 0.69, 95%CI = 0.49–0.99), FFM of the upper quartile (OR = 2.47, 95%CI = 1.50–4.07), and pre-pregnancy BMI (OR = 1.50, 95%CI = 1.13–1.99) remained predictors of birth weight more than 4 kg (Table 4). In contrast, no relationship was seen between odds of birth weight more than 4 kg and FM after adjustment for FFM.

**Table 4:** Adjusted Odds Ratios of Fat Free Mass Quartiles as Predictors of Birth Weight Greater Than 4 kg^α^

Included variables in model	Adjusted OR^b^	95% CI	*P*-value
Maternal age	0.69	0.49–0.99	0.041
Pre-pregnancy BMI	1.50	1.13–1.99	0.004
FFM ≥ 40.76 kg (Upper quartile)	2.47	1.50–4.07	<0.001

^a^Stepwise backwards elimination was used for selecting the variables included in the models

^b^Adjustments were made for maternal age, gestational age, BMI and FM

## Discussion

In our large prospective observational study of Han Chinese women with a singleton birth, the variables showed that the strong correlation with birth weight were placental weight, GWG, pre-pregnancy BMI, mid-pregnancy BMI, maternal FFM and FM during the first and second trimester. The variable only showed that the strong correlation with birth weight was placental weight and maternal FFM during the third trimester. The Pearson coefficient matrix showed maternal FFM in whole pregnancy process was correlated with birth weight. As shown in further multiple liner regression analysis in different trimesters respectively, we supported the conclusion that FFM were strong while FM did not contribute to the explanatory power in any significant way. 24% of the variation in birth weight was explained by FFM in the first trimester, 19% was by FFM, pre-pregnancy BMI and gestational age in the second trimester and12% of the variation explained by FFM. Our findings showed the birth weight was correlated with maternal FFM but not FM, which are consistent with those of previous smaller studies using BIA in pregnant women. Farah et al. reported that birth weight correlated with maternal FFM but not FM at 28 and 37 weeks of gestations [21]. Gernand et al. found that higher maternal FFM at 0–10 weeks of gestation were independently associated with higher birth weight [21]. Kent et al. also reported that birth weight correlated positively with maternal FFM in the first trimester but not adiposity [20]. In contrast, Forsum et al. suggested that FM both before pregnancy and in gestational week 32 was likely to be important for the increase in birth weight, together with gestational age at birth, PBF before pregnancy explained 45% of the variation in birth weight [17]. In addition, a longitudinal study of 169 women also found that maternal FFM during second trimester by using BIA was independently related to birth weight [18]. The contradictory conclusions of maternal body composition were maybe due to different sample, ethnic and testing time in previous studies.

After adjustment for age and gestational age, pre-pregnancy and mid-pregnancy BMI and FM, the increased maternal age became a protective factor (OR = 0.69) but the increased pre-pregnancy BMI (OR = 1.50) remained predictors of birth weight more than 4 kg. There was significantly associated with 2.47-fold increase in risk for birth weight more than 4 kg when FFM ≥ 40.76 kg (Upper quartile of FFM). Limited previous large-scale epidemiologic studies have examined the association between birth weight and maternal body fat composition. It is unclear why increased FFM and pre-pregnancy BMI were associated with higher birth weight. This could be due to the obesity status before pregnancy, rapid growth of the fetus in early second trimester and the increase of TBW in third trimester. From the 10^th^ week of gestation (8^th^ week of development), all major structures are already formed in the fetus, and they begin to grow rapidly. The growth of muscle tissue and bones plays a decisive role in birth weight. Thirteen to twenty-two weeks of gestation is a critical period: more muscle tissue and bones of fetus are developed, and the bones become harder [22]. In the third trimesters, the rapid growth of TBW plays an important role to increase of FFM, which was correlated with birth weight. Butte et al. also found that birth weight was correlated positively with gains in TBW and FFM but not in FM during 0–36 gestational weeks [10]. Lederman reported that FM of well-nourished women in late pregnancy did not contribute significantly to birth weight, but TBW did [13]. These inferences require further confirmed.

Our findings must be interpreted in the context of the study design and ethnicity. One of the strengths was that the information on both anthropometry measurements and pregnancy outcome collected through medical records rather than recall, which minimized potential recall bias. It is notable that women with pre-existing and emerging diseases which could influence birth weight were all excluded in our study. Nevertheless, our study does have some limitations. We used a foot-to-foot BIA instrument to analyze maternal body composition. Due to limited instrument, there is no way to acquire the data of TBW. Therefore we do not know whether or how this factor contributed to birth weight. Additionally, our findings showed the relationships among maternal body component indices and neonatal birth weight might vary among ethnic groups. The East Asians had lower BMI than other ethnic [23, 24]. We should be took more studies to prove this relationship among other ethnics or immigrants.

## Conclusions

Our key findings confirm the complex relationship between birth weight and maternal factors. The fact is that maternal FFM in whole pregnancy process was independently associated with increased offspring birth weight after controlling for other explanatory variables. These findings provide further evidence that maternal FFM but not FM may be important in programming intrauterine fetal weight growth. The interventions intended to reduce FM during pregnancy for non-diabetic women may not prevent LGA. The conclusion would highlight the feasibility of interventions to improve birth weight and decreasing obstetric complications.

## Abbreviations

BIA: Bioelectrical impedance analysis
BMI: Body mass index
FFM: Fat free mass
FFMI: Fat free mass index
FM: Fat mass
GDM: Gestational diabetes mellitus
GPMCCH: Center of prenatal care of the Gansu Provincial maternity & child care hospital
GWG: Gestational weight gain
LGA: Large for gestational age
OGTT: Oral glucose tolerance test
PBF: Percentage of body fat
TBW: Total body water
